# Immunohistochemical expression of CD10 in cutaneous basal and squamous cell carcinomas

**Published:** 2015

**Authors:** Shahriar Shafaei, Mahnaz Sharifian, Karimollah Hajian-Tilaki

**Affiliations:** 1 Department of Pathology, Faculty of Medicine, Babol University of Medical Sciences, Babol, Iran.; 2 Department of Biostatistics and Epidemiology, Faculty of Medicine, Babol University of Medical Sciences, Babol, Iran.

**Keywords:** CD 10, Immunostaining, BCC, SCC

## Abstract

**Background:**

Non-melanoma skin cancer is the most common malignant tumor in humans. The role of ultraviolet radiation is well-known in the pathogenesis of basal cell carcinoma (BCC) and squamous cell carcinoma (SCC). CD10 is a zinc-dependent metallopeptidase known as common acute lymphoblastic leukemia antigen (CALLA). Although CD10 expression has been investigated in some cutaneous tumors, to our knowledge, data regarding its expression in cutaneous epithelial neoplasms are very limited. In this study, we aimed to determine the immunohistochemical expression of CD10 in BCC and SCC and to find whether it could distinguish between these two skin malignancies.

**Methods:**

Twenty SCC and 42 BCC cases were retrieved randomly from Ayatollah Rouhani Hospital pathology archive and CD10 expression was determined in tumoral and stromal cells of each case based on immunohistochemical method. Positive CD10 staining was identified as brown cytoplasmic, with or without cell membrane staining.

**Results:**

In all the 20 SCC cases, tumor cells failed to stain with CD10 in contrast to the stromal cells that showed CD10 expression in 18 cases (90%). In BCC cases, the expression of CD10 was noted in tumor cells in 25 cases (59.5%) and in stromal cells of 32 cases (76.2%). There was no relation between CD10 expression in aggressive and non- aggressive BCC.

**Conclusion:**

Our findings suggest that CD10 is a useful immunohistochemical marker to differentiate between BCC and SCC. At least, if tumor cells were CD10 positive, this would favor BCC over SCC. Due to small number of aggressive BCC in contrast to non- aggressive types, more studies need to be done to prove or rule out this finding.

Basal-cell carcinoma (BCC) and squamous-cell carcinoma (SCC) are the most common tumors of skin cancer in human being which statistically constitutes 75% and 25% of non-melanoma skin cancers, respectively (1, 2)^.^ Typically, BCC occurs in the fourth decade of life and beyond particularly in the head and neck (3, 4). Basal cell carcinoma usually grows slowly with local invasiveness. There are several distinctive histopathologic types, namely, nodular, superficial, keratotic, pigmented, adenoid, morpheaform, micronodular, and basosquamous (5). The last three ones are considered as "aggressive types" of BCC due to their clinical behavior and the others are "nonaggressive" (6). Although BCCs virtually never metastasize, there have been a few reports in the literature of BCCs that developed metastases, and the incidence has been estimated to be 1: 1000 to 1:35 000.

Well-differentiated SCC generally arises in the setting of epidermal changes consistent with actinic keratosis. Histopathologically, there is a downward proliferation of lobules and detached aggregations of glassy, brightly eosinophilic keratinocytes containing nuclei with some degree of pleomorphism and mitoses. Intercellular bridges (desmosomes) are often apparent, along with keratin pearls and apoptotic cells. Poorly differentiated SCCs display progressive and overlapping features, ending in highly infiltrative tumors that lack overt keratinization and sometimes have a spindle cell morphology (7). Generally SCC of the skin has more potential to develop metastatic disease than BCC (7).

The distinction between BCC and SCC is usually made readily on the basis of defined histological criteria mentioned above. But some specimens reveal inherently ambiguous morphology that make it difficult to classify them as BCC or SCC based on H&E staining. In this condition, the utility of IHC methods could be helpful. Such distinction is clinically important because the risk of progressive disease is significantly higher with SCC than BCC (6).

CD10 which is a zinc-dependent metallopeptidase known as common acute lymphoblastic leukemia antigen (CALLA) is a useful marker in the classification and diagnosis of leukemia/ lymphoma. CD10 has been reported in both epithelial (bladder, hepatocellular, renal cell) carcinomas (6, 8, 9) and mesynchymal neoplasms (endometrial stromal sarcoma of the uterus) (10). In normal skin, CD10 immunostainting is present in sebaceous glands, periadnexal dermis, and inner root sheath cells of vellus hair follicles and occasional endothelial cells (11-13). CD10 has a potential role in differentiating cutaneous metastatic renal cell carcinoma from adnexal neoplasm with eccrine and apocrine differentiation but not sebaceous differentiation (14). CD10 expression has been suggested to be helpful in distinguishing between atypical fibroxanthomas (strong diffuse expression in 94% of cases) and SCC (weak and patchy expression in 50% of cases) (14). It has been recognized as a useful marker for differentiating BCC from trichoepithelioma (TE) (15). However, the data regarding its expression in cutaneous epithelial neoplasms such as BCC and SCC are very limited. It has been claimed in some recent articles that CD10 can be useful as a specific marker to distinguish BCC from SCC (16). Thus, the aim of this study was to determine the immunohistochemical expression of CD10 in BCC and SCC to see if the difference exists and also to find out whether it could be useful in distinguishing BCC from SCC in situation that we have difficulties with H&E staining.

## Methods

This is a descriptive-analytical cross-sectional study in which 42 cases of BCC and 20 SCC of excisional or punch biopsy specimens were randomly retrieved from surgical pathology archive of Ayatollah Rouhani Hospital within 2013. Patients’ data including sex, age and final diagnosis were recorded. Hematoxylin & eosin (H&E) slides were reviewed microscopically and only those with definite and characteristic patterns for BCC or SCC were included in the study. BCC subtypes were determined and then we classified the subtypes in two major groups of "aggressive" and "non-aggressive" which was explained above. Grading of SCC was performed in the most aggressive area and was divided into well, moderately and poorly differentiated. From each representative block, 3-mm-thick sections were cut and mounted on poly-L-lysine – pre-coated slides for immunostaining of CD10. Slides were dried for 60min in an oven at 60˚C. Deparaffinization and rehydration were done and after antigen retrieval and inactivation of endogenous peroxidase, the sections were stained with antibody of CD10 (clone 56C6, RTU-CD10-270 Novacastra) .Normal tonsil was used as positive control.

CD10 positivity was considered as brown cytoplasmic and/or membrane staining. Ten high power fields were examined for each case of BCC and SCC, then the mean percentage of positive cells in tumoral (epithelial components) and/or stromal (mesynchymal components around epithelial cells) cells were calculated as follows <10% as negative, 10-50% as low expression and >50% as high expression (17). The data were collected, tabulated and statistically analyzed with ''statistical package for the social sciences (SPSS), Version 22''. Chi-square test was applied to compare the proportion of CD10 expression between BCC and SCC and p-value less than 0.05 was considered as statistically significant.

## Results

Our study included 42 cases of BCC with age ranging from 42- 81 years, with mean 58.64±10.03 years. Twenty nine (69%) cases were males and 13 (31%) females. Ninety-two percent of the lesions were diagnosed in sun exposed area and 8% were in covered area. Histopathological examination revealed 21 cases (50%) as solid subtype, 9 adenoid, 3 superficial, 2 morpheaform, 2 micronodular, 4 pigmented and 1 keratotic. In this study, we had 4 cases of aggressive BCC (2 morpheaform, 2 micronodular) and 38 cases of non-aggressive types (the other subtypes). This study also included 20 cases of SCC. Their ages ranged from 53-80 years, with mean (±SD) 64.25±7.77. Twelve cases (60%) were males. About 45% of the lesions were diagnosed in lower lip area. Histoplathologic examination revealed 75% cases of well-differentiated SCC, 20% cases of moderately differentiated and the rest were poorly differentiated. In the BCC group, CD10 expression in tumoral cells was detected in 25 out of 42 cases (35.7% with low expression and 23.8% with high expression) whereas stromal cells were positive in 76.2% of cases (31% with low expression and 45.2% with high expression). Also, CD10 expression in aggressive and non-aggressive BCC types was shown in table1.

**Table 1:** Comparison of CD10 expression in aggressive and non-aggressive BCC types

	**Stromal cells**			**Tumoral cells**		**Component**
**High** **No(%)**	**Low** **No(%)**	**Negative** **No(%)**	**High** **No(%)**	**Low** **No(%)**	**Negative** **No(%)**	
16 (42.1)	12 (31.5)	10 (26.3)	9 (23.6)	14 (36.8)	15 (39.4)	Non aggressive BCC (38 case)
3 (75.0)	1 (25.0)	0 (0.0)	1 (25.0)	1 (25.0)	2 (50.0)	Aggressive BCC (4 case)

In nearly all SCC cases (90%), CD10 immunopositivity was noted in stromal cells (55% with low expression, 35% with high expression) but none of them were immunoreactive for CD10 in tumoral cells. Comparing SCC and BCC groups with regard to CD10 expression, there was a significant difference between CD10 expression in tumoral cells (P=0.001) but not in stromal cells (P=0.157) in both groups (table 2). There was no statistically significant association in CD10 positivity of SCC or BCC cases with age, sex and subtypes in either tumoral or stromal cells.

**Table 2:** Clinical and immunohistochemical comparison between BCC and SCC cases

	**BCC(n=42)** **No(%)**	**SCC(n=20)** **No(%)**	**P value**
Tumor cells Positive Negative	25 (59.5) 17 (40.5)	0 (0.0) 20 (100)	0.001
Stromal cells Positive Negative	22 (76.2) 10 (23.8)	18 (90.0) 2 (10.0)	0.157

## Discussion

Our findings show that there is a significant difference between CD10 expression in tumoral cells in SCC and BCC (P=0.001). The tumoral cells in all SCC cases were completely negative for CD10 that is similar to several previous reports both in skin and in oral cavity SCC (18-19). In our study, also 59.5% of BCC cases, were positive for CD10 in tumoral cells. These results are in accordance with those of previous reports in tumoral cells of BCC and SCC, respectively (17, 19-22). 

We also observed a significant difference between CD10 expression in tumoral cells of SCC and BCC (P=0.001). Our results reflect the fact that BCC is biologically different from SCC. We assume that in addition to the well-defined histological criteria, CD10 might be a useful immunohistochemical marker, in difficult cases, to differentiate between BCC and SCC. At least, if tumor cells were CD10 positive, this would favor BCC over SCC. A recent study has reported that CD10 was strongly expressed in 14 out of 14 superficial BCCs and failed to be expressed in 13 out of 13 superficially invasive SCC and SCC in situ (20). 

These findings strongly support our results and suggest the utility of CD10 in the differentiation between BCC and SCC. In our study of the stromal cells in BCC, 76.2% of cases were positive for CD10 while in SCC cases 90% were positive. We did not observe a significant difference between CD10 expression in stromal cells of SCC and BCC. Our results of CD10 expression in stromal cells of BCC and SCC, respectively are in accordance with Aiad et al (19). Whereas it is not similar to Aslani et al (20). And Heidarpour et al (21). As the later studies found statistically difference between stromal cells in BCC and SCC. Our results revealed that there was no statistically significant association in CD10 positivity of aggressive and non-aggressive BCC cases in either tumoral or stromal cells in this study but Yada et al. (17). Found that sclerosing BCC tended to have more frequent CD10 – immunopositive stromal cells than other types. He also revealed that the absence of CD10 in cutaneous epithelial tumoral cells and its overexpression in the stromal cells might be associated with an invasive capacity of tumors.

In Conclusion, Our findings suggest that CD10 is a useful immunohistochemical marker to differentiate between BCC and SCC. At least, if tumor cells were CD10 positive, this would favor BCC over SCC. There was no relation between CD10 expression in aggressive and non-aggressive BCC. Due to a small number of aggressive BCC in contrast to non-aggressive types, more studies need to be done to prove or rule out this result.

## Notes

**Funding:** This study was funded by the Vice-Chancellery for Research and Technology of Babol University of Medical Sciences, Babol, Iran
